# Raddeanin A augments the cytotoxicity of natural killer cells against chronic myeloid leukaemia cells by modulating MAPK and Ras/Raf signalling pathways

**DOI:** 10.1111/jcmm.70016

**Published:** 2024-08-22

**Authors:** Ming‐Ju Hsieh, Jen‐Tsun Lin, Yi‐Ching Chuang, Yu‐Sheng Lo, Chia‐Chieh Lin, Hsin‐Yu Ho, Mu‐Kuan Chen

**Affiliations:** ^1^ Oral Cancer Research Center Changhua Christian Hospital Changhua Taiwan; ^2^ Doctoral Program in Tissue Engineering and Regenerative Medicine, College of Medicine National Chung Hsing University Taichung Taiwan; ^3^ Graduate Institute of Clinical Medicine, College of Medicine National Chung Hsing University Taichung Taiwan; ^4^ Graduate Institute of Biomedical Sciences China Medical University Taichung Taiwan; ^5^ Division of Hematology and Oncology, Department of Medicine Changhua Christian Hospital Changhua Taiwan; ^6^ Department of Otorhinolaryngology, Head and Neck Surgery Changhua Christian Hospital Changhua Taiwan; ^7^ Department of Post‐Baccalaureate Medicine, College of Medicine National Chung Hsing University Taichung Taiwan

**Keywords:** granzyme B, MAPK signalling, natural killer cells, Raddeanin A

## Abstract

Natural killer (NK) cell therapy, a developing approach in cancer immunotherapy, involves isolating NK cells from peripheral blood. However, due to their limited number and activity, it is essential to significantly expand these primary NK cells and enhance their cytotoxicity. In this study, we investigated how Raddeanin A potentiate NK activity using KHYG‐1 cells. The results indicated that Raddeanin A increased the expression levels of cytolytic molecules such as perforin, granzymes A and granzymes B, granulysin and FasL in KHYG‐1 cells. Raddeanin A treatment increased CREB phosphorylation, p65 phosphorylation, NFAT1 and acetyl‐histone H3 expression. Raddeanin A elevated caspase 3 and PARP cleavage, increased t‐Bid expression, promoting apoptosis in K562 cells. Furthermore, it reduced the expression of HMGB2, SET and Ape1, impairing the DNA repair process and causing K562 cells to die caspase‐independently. Additionally, Raddeanin A increased ERK, p38 and JNK phosphorylation at the molecular level, which increased granzyme B production in KHYG‐1 cells. Raddeanin A treatment increased Ras, Raf phosphorylation, MEK phosphorylation, NKG2D, NKp44 and NKp30 expression in KHYG‐1 cells. Collectively, our data indicate that Raddeanin A enhances the cytotoxic activity of NK cells against different cancer cells.

## 1 INTRODUCTION

Raddeanin A is a naturally occurring triterpenoid saponin primarily found in the roots of *Anemone raddeana* Regel,^1^ a perennial herbaceous plant native to China and Mongolia.^2^, ^3^ This bioactive compound has garnered significant attention in scientific research due to its diverse pharmacological properties and potential therapeutic applications. Raddeanin A possesses a unique chemical structure characterized by a triterpene backbone with attached sugar moieties, rendering it a member of the saponin family.^4^ Recent studies have provided compelling evidence showcasing the potential of Raddeanin A as a potent anti‐cancer agent. Investigations have revealed its ability to impede the proliferation, induce apoptosis and suppress invasion in a spectrum of human tumour cells. Among these, hepatocellular carcinoma cells, gastric cancer cells and non‐small‐cell lung carcinoma cells have been particularly studied.^5^, ^6^, ^7^ Raddeanin A has therefore become a highly intriguing drug development prospect and research into its mechanisms of action and possible therapeutic benefits on human natural killer (NK) leukaemia cells has gained a great deal of attention.

NK cells have cytotoxic granules such as PRF1, GZMs and granulysin, which stands out as a key player in NK‐mediated cytotoxicity. After reaching target cells, NK cells eliminate target cells by activating death ligands (FasL, TRAIL) or/ and by releasing cytotoxic granules.^8^, ^9^ Our earlier investigation demonstrated that Shuterin potentiated the cytolytic activity of the human leukaemic NK cell line KHYG‐1 by via MAPK and Ras/Raf signalling pathways.^10^ KHYG‐1 cell line is often used in research related to NK cells due to its characteristics resembling mature NK cells.^11^ In this present we explored the mechanism by how Raddeanin A potentiate NK activity in KHYG‐1 cells.

## 2 MATERIALS AND METHODS

### 2.1 Chemicals and reagents

**FIGURE 1:**
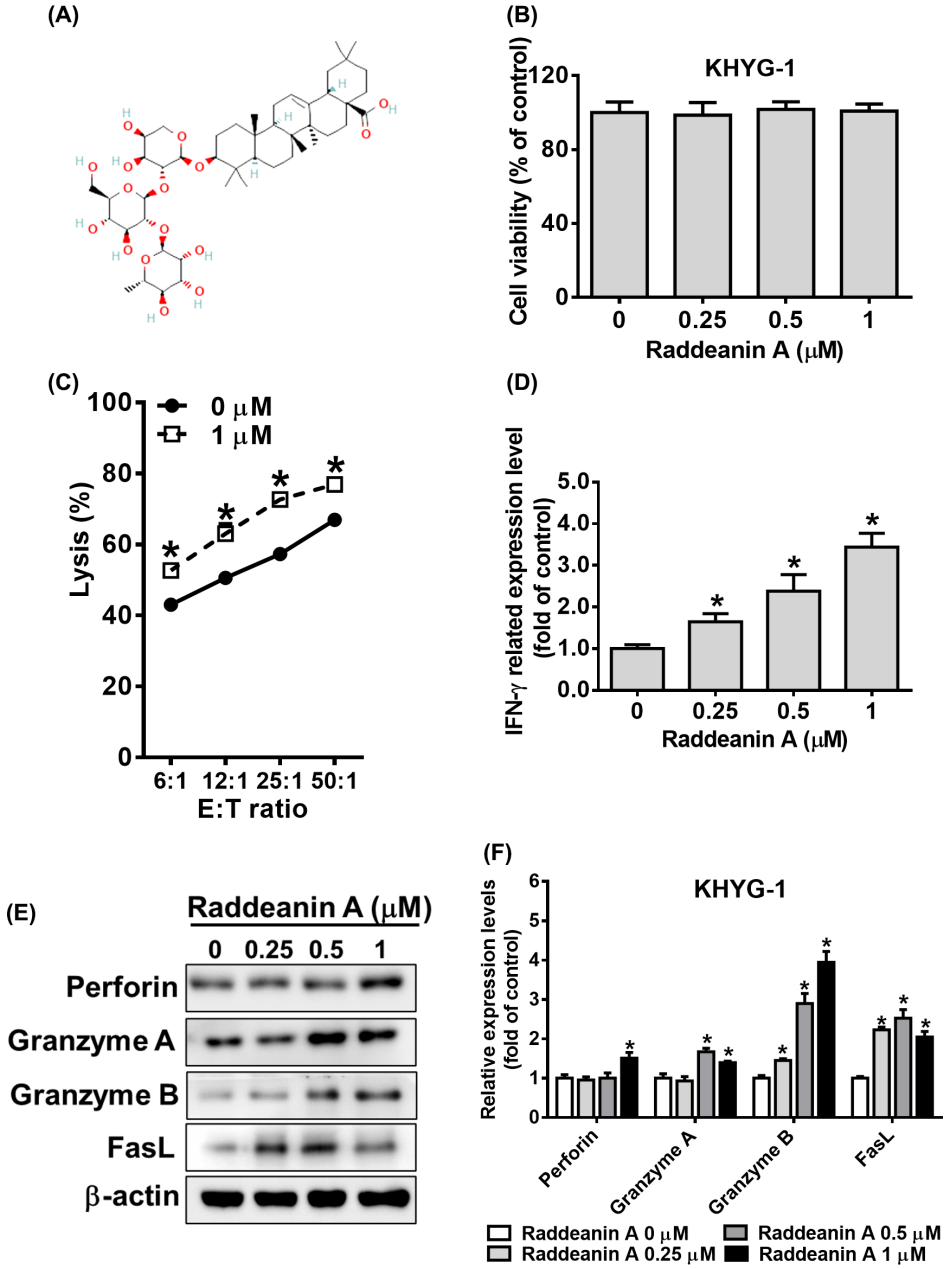
Raddeanin A activates the cytotoxicity effect in KHYG‐1 cells. (A) Chemical structure of Raddeanin A. (B) KHYG‐1 cells were treated with Raddeanin A for 72 h and then subjected to WST‐8 assays for cell viability assessment. (C) Cytolytic effects of KHYG‐1 cells treated with (or without) Raddeanin A for 72 h on K562 cells. Cytotoxicity was determined through the Calcein AM release assay. (D) Levels of IFN‐γ in the culture supernatants of KHYG‐1 cells treated with the indicated dose of Raddeanin A for 24 h, then measured through enzyme‐linked immunosorbent assay (ELISA). (E) After 24 h of Raddeanin A treatment, the cytotoxic effectors of the KHYG‐1 cells were analysed through western blotting. (F) ImageJ was used for protein quantification; the levels of all proteins were normalized to that of β‐Actin. Data were presented as the mean ± standard deviation (*n* = 3) of three independent experiments. *p < 0.05 compare with control.

Raddeanin A (purity ≥98%) (Figure 1A) was obtained from ChemFaces (Wuhan, China) and calcein AM was purchased from AAT Bioquest (Pleasanton, CA, USA). The stock solutions of Raddeanin A and calcein AM were prepared in dimethyl sulfoxide (DMSO) and stored at −20°C. Other chemical reagents used in this study including histone deacetylase inhibitor (trichostatin A [TSA]), granzyme B inhibitor (Z‐AAD‐CMK), protease inhibitor cocktail and phosphatase inhibitor cocktail, were purchased from Sigma‐Aldrich (St Louis, MO, USA). MAPK pathway inhibitors for protein kinase B (AKT; LY‐294002), extracellular signal‐regulated kinase (ERK) 1/2 (U0126), c‐Jun N‐terminal kinase (JNK; SP‐600125) and P38 (SB‐203580) were purchased from Santa Cruz Biotechnology (Santa Cruz, CA, USA).

### 2.2 Cell culture

Human NK leukaemia cell line KHYG‐1 (Japanese Collection of Research Bioresources [JCRB] accession no. JCRB0156) and human chronic myelogenous leukaemia cell line K562 (JCRB0019) were obtained from JCRB (Japan). KHYG‐1 cells were cultured in Roswell Park Memorial Institute (RPMI‐1640) (Life Technologies, Grand Island, NY, USA) medium supplemented with 10% fetal bovine serum (FBS) (Merck Millipore, Burlington, MA, USA), 100 U/mL recombinant human interleukin (IL)‐2 (Cat. #200–02; PeproTech, Cranbury, NJ, USA), 100 U/mL penicillin G and 100 μg/mL streptomycin sulfate. K562 cells were cultured in the same medium without IL‐2 supplementation. The cultured cells were incubated at 37°C under 5% CO_2_. NK cells were isolated from the peripheral blood of adult donors with head and neck squamous cell carcinoma (HNSCC). Peripheral blood mononuclear cells were cultured using BINKIT (Biotherapy Institute of Japan, Tsukuba, Japan), as per the manufacturer's instructions. The subculture medium comprised 10% heat‐inactivated autologous plasma and 400 U/mL recombinant human IL‐2. All experiments were performed within day 21 of culture.

### 2.3 Cell proliferation assay

The effects of Raddeanin A on the proliferation of KHYG‐1, K562, NPC‐039 and FaDu cells were evaluated through WST‐8 assays (Beyotime Technology, Shanghai, China). The cells were seeded onto 96‐well plates (5 × 10^4^ cells/well), treated with different doses of Raddeanin A (0.25, 0.5 and 1 μM), and cultured with or without IL‐2 for 24 or 72 h. Untreated cells were used as experimental controls. Finally, the absorbance was measured at 450 nm to assess cell proliferation.

### 2.4 Cell cytotoxicity assay

Cell cytotoxicity was assessed using calcein AM. K562 cells cultured in complete RPMI 1640 media were stained with 10 μM calcein AM for 30 min at 37°C under 5% CO_2_. The cells (target cell) were then seeded onto 96‐well plates. KHYG‐1 cells (effector cells) were treated with indicated doses of Raddeanin A for 72 h. Then, KHYG‐1 and K562 cells were cocultured for 2 h (without Raddeanin A). Fluorescence intensity was measured at the excitation/mission wavelength of 485/530 nm. Percent lysis was calculated using the following formula: [(experimental release − spontaneous release)/(maximum release − spontaneous release)] × 100%.

### 2.5 Cytokine secretion

KHYG‐1 cells were centrifuged at 190 *g* for 5 min at 4°C. The supernatant was obtained to determine the levels of IFN‐γ using LEGEND MAX Human IFN‐γ ELISA Kit (BioLegend, San Diego, CA), as per the manufacturer's instructions.

### 2.6 Western blotting

Western blotting was performed as per a previously described method. In brief, KHYG‐1, K562, NPC‐039 and FaDu cells were treated with indicated doses of Raddeanin A for 24 h and lysed using radioimmunoprecipitation assay buffer. The lysates were separated through sodium dodecyl sulfate polyacrylamide gel electrophoresis and transferred onto polyvinylidene fluoride membranes (Merck Millipore). The membranes were incubated with 5% skim milk for 1 h, followed by incubation with appropriate primary antibodies overnight at 4°C (antibodies against perforin #62550, granzyme A #4928, granzyme B #17215, FasL #68405, Fas #4233, p‐CREB #9198, CREB #9197, p‐p65 #3033, p65 #8242, acetyl‐histone H3 #9677, histone H3 #4499, HMGB2 #14163, Ape1 #10519 c‐PARP #9542, c‐caspase 9 #9502, c‐caspase 8 #9496, c‐caspase 3 #9664, t‐bid #2002, p‐AKT #4060, AKT #2920, p‐ERK #4370, ERK #4695, p38 #9212, p‐JNK #4668, JNK #9258, β‐actin #4970, Ras #3965, p‐Raf #9427, Raf #9422, p‐MEK #9154 and MEK #9126 were purchased from Cell Signalling Technology, Danvers, MA, USA; antibodies against p‐p38 #44‐684G, NKG2D #PA5‐109890, NKp44 #PA5‐119302 and NKP30 #PA5‐96595 were purchased from Invitrogen, Carlsbad, MA, USA; antibody against SET #NBP1‐33713 was purchased from Novus Biologicals, Centennial, CO, USA). Subsequently, peroxidase‐conjugated secondary antibodies were added to the membranes and incubated for 60 min. The membranes were then subjected to ECL detection and photographed using chemiluminescence fluorescence ImageQuant biomolecule imaging system (ChemiDoc MP (Bio‐Rad)). ImageJ was used for protein quantification.

### 2.7 Mitochondrial membrane potential measurement

Mitochondrial membrane potential assay was described previously.^12^ Briefly, KHYG‐1 cells were treated with indicated doses of Raddeanin A, and the treated cells were added to the upper wells of a Transwell insert (Greiner Bio‐One, Monroe, NC, USA). K562 cells (in RPMI media) were added to the lower wells of the same insert and incubated according to previously described coculture methods.^13^ The effector cells were cocultured with the target cells at an effector: target (E: T) ratio of 6:1 for 24 h at 37°C under 5% CO_2_. After treatment with Raddeanin A, the cells were collected and stained with Muse MitoPotential dye (Merck Millipore) for 20 min at 37°C, followed by incubation with 7‐AAD for 5 min and measurement of experimental signals by Muse Cell Analyser flow cytometry (Merck Millipore), and the data were analysed by Muse Cell Soft V1.4.0.0 Analyser.

### 2.8 Annexin V/PI double staining

The Annexin V/PI double stain of apoptosis detection was described previously.^14^ KHYG‐1 cells were treated with indicated doses of Raddeanin A and added to the upper wells of a Transwell insert (Greiner Bio‐One). K562 cells (in RPMI media) were added to the lower wells of the same insert and incubated according to previously described coculture methods.^13^ The effector cells were cocultured with the target cells at an effector: target (E: T) ratio of 6:1 for 24 h at 37°C under 5% CO_2_. After treatment with Raddeanin A, the cells were collected and resuspended in 100 μL PBS with 2% BSA. Annexin V/PI double stain (Merck Millipore) was added to the cell suspension and incubated for 20 min at room temperature in the dark. Cell apoptosis stage was measured by Muse Cell Analyser flow cytometry (Merck Millipore), and the data were analysed by Muse Cell Soft V1.4.0.0 Analyser.

### 2.9 Statistical analysis

Each experiment was performed at least thrice. The data are presented as mean ± standard deviation. One‐way analysis of variance and Student's *t* tests were performed. A *p* value of <0.05 indicated statistical significance. Sigma‐Stat 2.0 (Jandel Scientific, San Rafael, CA, USA) was used for the data analysis.

## 3 RESULTS

### 3.1 Effect of Raddeanin A on KHYG‐1cells

KHYG‐1 cells were treated with different concentration of Raddeanin A (0.25, 0.5 and 1 μM) for 72 h and analysed for cell viability using to WST‐8 assay. No significant changes were observed between control and treatment group (Figure 1B). Cytolytic effects of KHYG‐1 cells treated with (or without) Raddeanin A for 72 h. As shown in Figure 1C, Raddeanin A treatment greatly increased cytotoxicity in KHYG‐1 cells at 1 μM concentration. IFN‐γ secretion was assessed in Raddeanin A‐treated cells. The therapy boosted IFN‐γ secretion based on dosage (Figure 1D). Raddeanin A was tested for its influence on perforin, granzyme A, granzyme B, FasL and IFN‐γ transcriptional activation using western blotting. Raddeanin A treatment increased perforin, granzyme A, granzyme B and FasL protein expression (Figure 1E,F).

### 3.2 Raddeanin A alters CREB, p65, NFAT1 and acetyl histone expression

**FIGURE 2:**
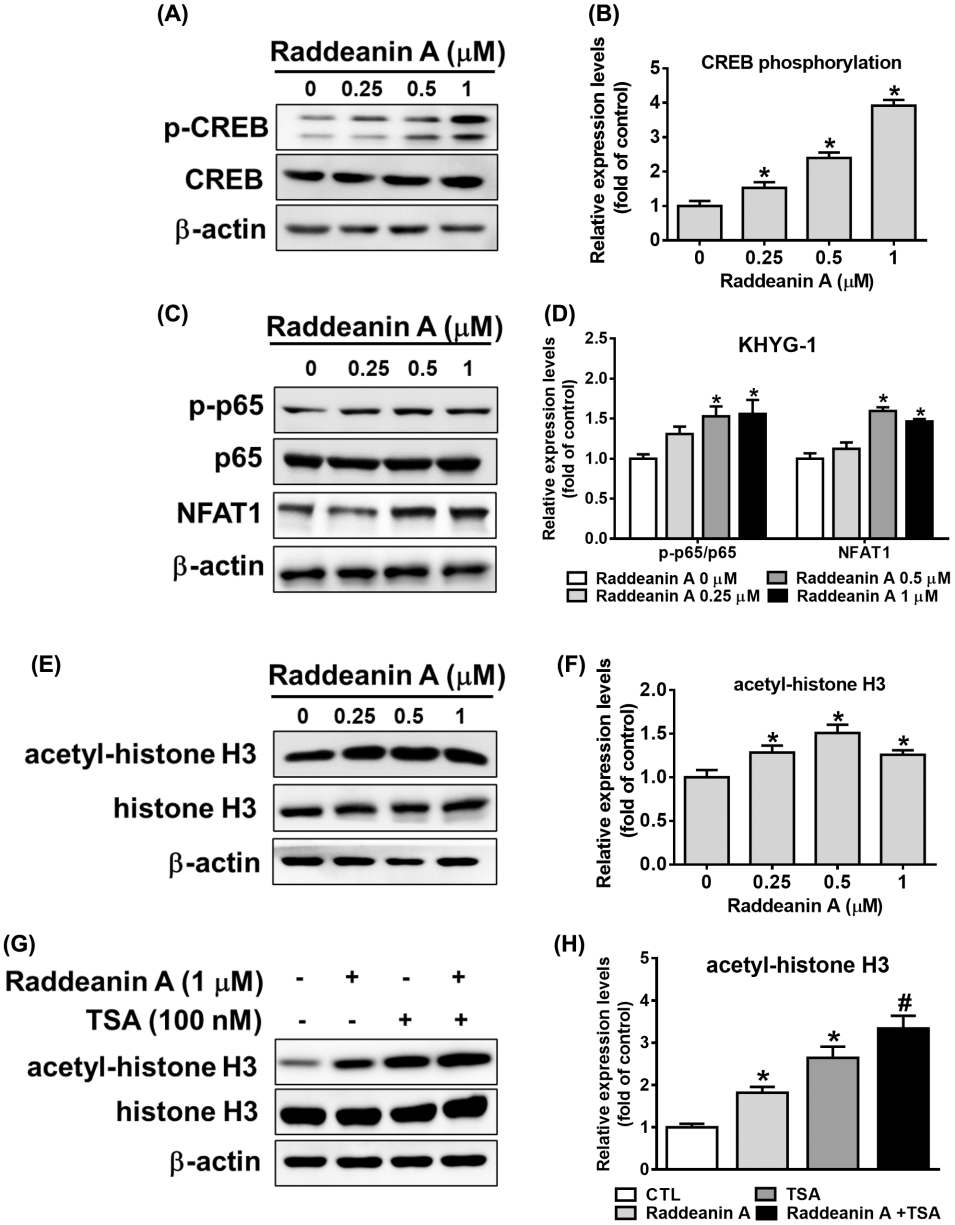
Effects of Raddeanin A on CREB, p65, NFAT1 and acetyl histone activity in KHYG‐1 cells. (A–F) The cells were treated with Raddeanin A for 24 h. Protein levels of CREB phosphorylation/CREB, p65 phosphorylation/p65, NFAT1 and acetyl‐histone H3/histone H3. (G, H) KHYG‐1 cells were pretreated with TSA for 1 h or left untreated; subsequently, the cells were treated with Raddeanin A for 24 h. Protein levels of acetyl‐histone H3/ histone H3. ImageJ was used for protein quantification; the levels of all proteins were normalized to that of β‐Actin. Data were presented as the mean ± standard deviation (*n* = 3) of three independent experiments. *p < 0.05 compare with control; # p < 0.05 compare with TSA alone.

Granzyme B expression can be influenced by the transcriptional activity regulated by CREB, p65 and NFAT1.^15^, ^16^ To investigate whether Raddeanin A regulates granzyme B expression, we treated KHYG‐1 cells with Raddeanin A and subsequently examined the phosphorylation of CREB, p65, as well as the levels of NFAT1. Raddeanin A treatment increased CREB phosphorylation, p65 phosphorylation and NFAT1 expression in the KHYG‐1 cells (Figure 2A–D). Raddeanin A markedly increased acetyl‐histone H3 (Lys9/Lys14) expression, allowing transcription factors such as CREB, p65 and NFAT1 to access DNA and promote gene transcription. (Figure 2E,F). Cells pretreated with TSA dramatically increased acetyl histone H3 expression in comparison to the control group (Figure 2G,H).

### 3.3 Effects of Raddeanin A on caspase mediated apoptosis

**FIGURE 3:**
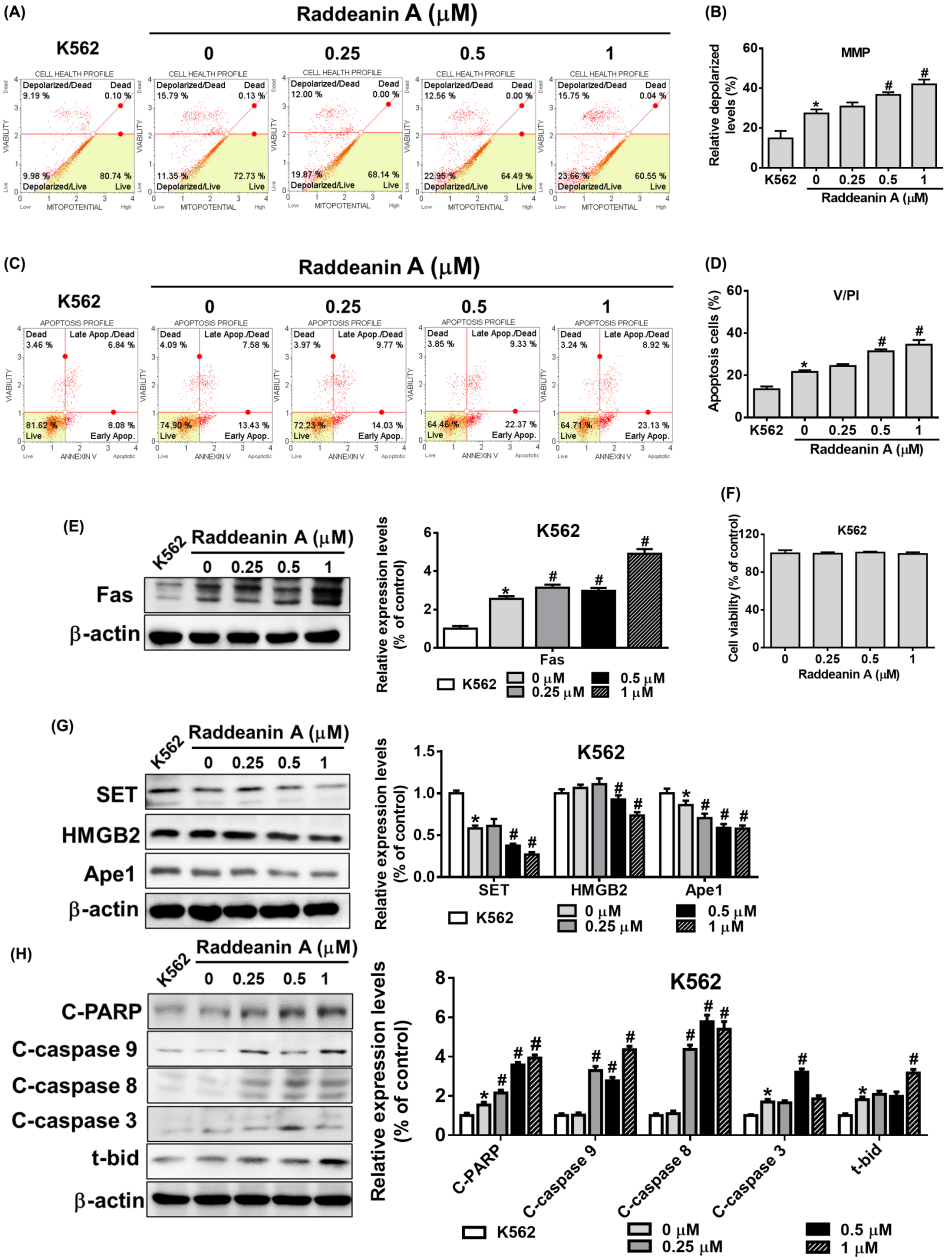
Raddeanin A‐treated NK cells enhance apoptosis in K562 cells. Raddeanin A induced apoptosis in cocultured K562 cells. KHYG‐1 cells were pretreated with Raddeanin A for 24 h before being transferred to a co‐culture system. (A and B) Mitochondrial membrane potential assay and (C and D) annexin V/PI stain assay of K562 cells were measured after Raddeanin A treatment by Muse Cell Analyser. Quantitative data were analysed by Muse Cell Software V1.4.0.0. (E) Fas protein levels of Raddeanin A‐induced apoptosis. K562 cells were cocultured with KHYG‐1 cells at an effector: Target ratio of 6:1 And then treated with Raddeanin A for 24 h. (F) K562 cells were treated with Raddeanin A for 24 h. Cell viability was assessed using WST‐8 assays. (G, H) Protein levels of Raddeanin A‐induced apoptosis. K562 cells were cocultured with KHYG‐1 cells at an effector: Target ratio of 6:1 And then treated with Raddeanin A for 24 h. ImageJ was used for protein quantification; the levels of all proteins were normalized to that of β‐Actin. Data were presented as the mean ± standard deviation (*n* = 3) of three independent experiments. *p < 0.05 compare with control; # p < 0.05 compare with cotreatment control.

Based on the observed enhancement of KHYG‐1 cell cytotoxicity against K562 cells by Raddeanin A, we proceeded to examine the impact of Raddeanin A treatment on apoptotic pathways in K562 cells co‐cultured with KHYG‐1 cells. Raddeanin A treatment dose‐dependently enhanced depolarized cells (Figure 3A,B). Similarly, treatment with Raddeanin A enhanced apoptotic cells in early and late stages. After being treated with Raddeanin A (0.25–1.0 μM) for 24 hours, the overall apoptotic cell percentage of the control group (K562 cells co‐cultured with KHYG‐1 cells) increased to 23.8%, 31.7% and 32.05%, respectively (Figure 3C,D).

Next, we assessed the impact of Raddeanin A on the expression of apoptotic proteins in cocultured K562 cells. Raddeanin A did not impact the viability of K562 cells (Figure 3F). The Raddeanin A treatment dramatically raised the expression of Fas and lowered SET, Ape1 and HMGB2 expressions in K562 cells, as shown in Figure 3E,G. Furthermore, as shown in Figure 3H, Raddeanin A increased t‐Bid, cleaved caspase 9, 3, 8 and cleaved PARP expression. These findings suggest that Raddeanin A may initiate both caspase‐dependent and independent cell death pathways in cocultured K562 cells, hence causing apoptosis.

**FIGURE 4:**
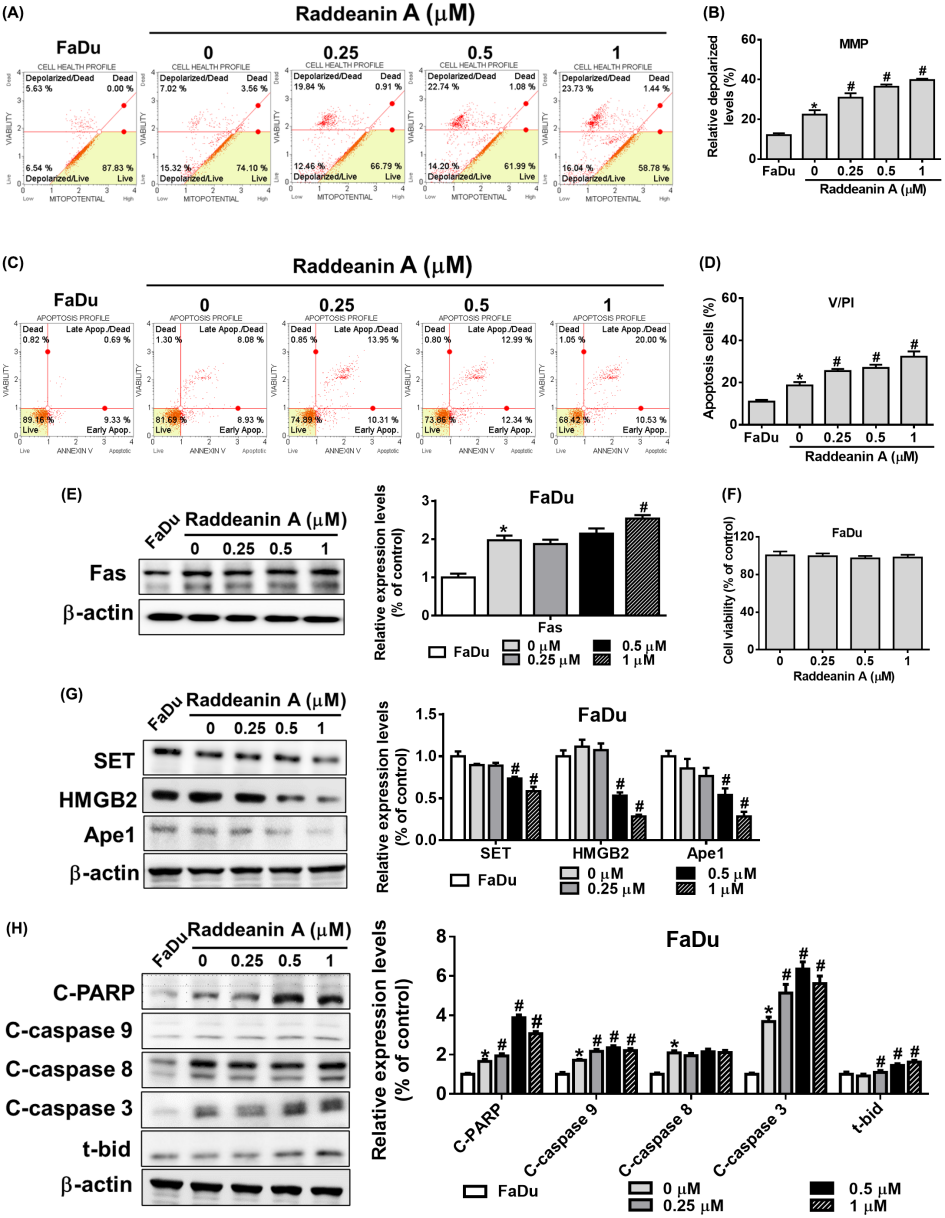
Raddeanin A‐treated NK cells enhance apoptosis in FaDu cells. (A, B) Mitochondrial membrane potential assay and (C, D) annexin V/PI stain assay of FaDu cells were measured after Raddeanin A treatment by Muse Cell Analyser. Quantitative data were analysed by Muse Cell Software V1.4.0.0. (E) Fas protein levels of Raddeanin A‐induced apoptosis. FaDu cells were cocultured with KHYG‐1 cells at an effector: Target ratio of 6:1 And then treated with Raddeanin A for 24 h. (F) FaDu cells were treated with Raddeanin A for 24 h. Cell viability was assessed using WST‐8 assays. (G, H) Protein levels of Raddeanin A‐induced apoptosis. FaDu cells were cocultured with KHYG‐1 cells at an effector: Target ratio of 6:1 and then treated with Raddeanin A for 24 h. ImageJ was used for protein quantification; the levels of all proteins were normalized to that of β‐Actin. Data were presented as the mean ± standard deviation (*n* = 3) of three independent experiments. *p < 0.05 compare with control; # p < 0.05 compare with cotreatment control.

**FIGURE 5:**
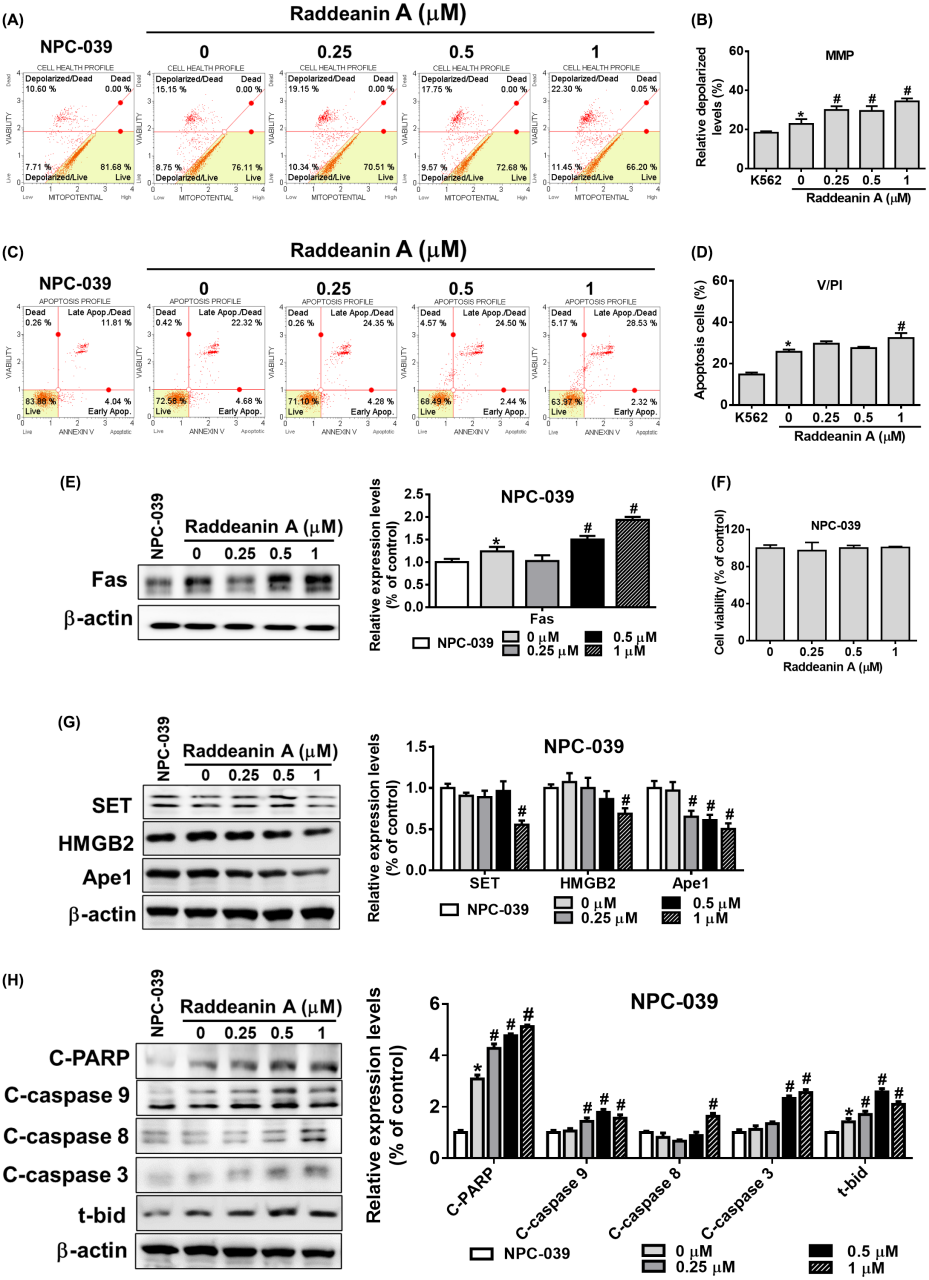
Raddeanin A‐treated NK cells enhance apoptosis in NPC‐039 cells. (A, B) Mitochondrial membrane potential assay and (C, D) annexin V/PI stain assay of NPC‐039 cells were measured after Raddeanin A treatment by Muse Cell Analyser. Quantitative data were analysed by Muse Cell Software V1.4.0.0. (E) Fas protein levels of Raddeanin A‐induced apoptosis. NPC‐039 cells were cocultured with KHYG‐1 cells at an effector: Target ratio of 6:1 And then treated with Raddeanin A for 24 h. (F) NPC‐039 cells were treated with Raddeanin A for 24 h. Cell viability was assessed using WST‐8 assays. (G, H) Protein levels of Raddeanin A‐induced apoptosis. NPC‐039 cells were cocultured with KHYG‐1 cells at an effector: Target ratio of 6:1 And then treated with Raddeanin A for 24 h. ImageJ was used for protein quantification; the levels of all proteins were normalized to that of β‐Actin. Data were presented as the mean ± standard deviation (*n* = 3) of three independent experiments. *p < 0.05 compare with control; # p < 0.05 compare with cotreatment control.

We carried out comparable studies with the FaDu and NPC‐039 cell lines to see if these effects of Raddeanin A are unique to cancer patients. Raddeanin A‐treated FaDu cells and Raddeanin A‐treated NPC‐039 cells showed a substantial increase in mitochondrial membrane depolarization and apoptosis (Figure 4A–D) (Figure 5A–D), which was similar to what was seen in K562 cells (Figure 3). Though, no cell death was observed in Raddeanin A treated FaDu cells (Figure 4F) and Raddeanin A treated NPC‐039 cells (Figure 5F). Furthermore, Raddeanin A treatment increased Fas, cleaved PARP, cleaved caspase 3, 9, 8 and t‐Bid expression and decreased SET, Ape1 and HMGB2 expression in cocultured FaDu cells (Figure 4E,G,H) and in cocultured NPC‐039 cells (Figure 5E,G,H). Out study shows that, Raddeanin A treated KHYG‐1 cells have the capability to induce cytotoxic effects on FaDu and NPC‐039 cancer cells as well, indicating a potential broader application of Raddeanin A in cancer treatment.

### 3.4 Effect of Raddeanin A on cell signalling pathways

**FIGURE 6:**
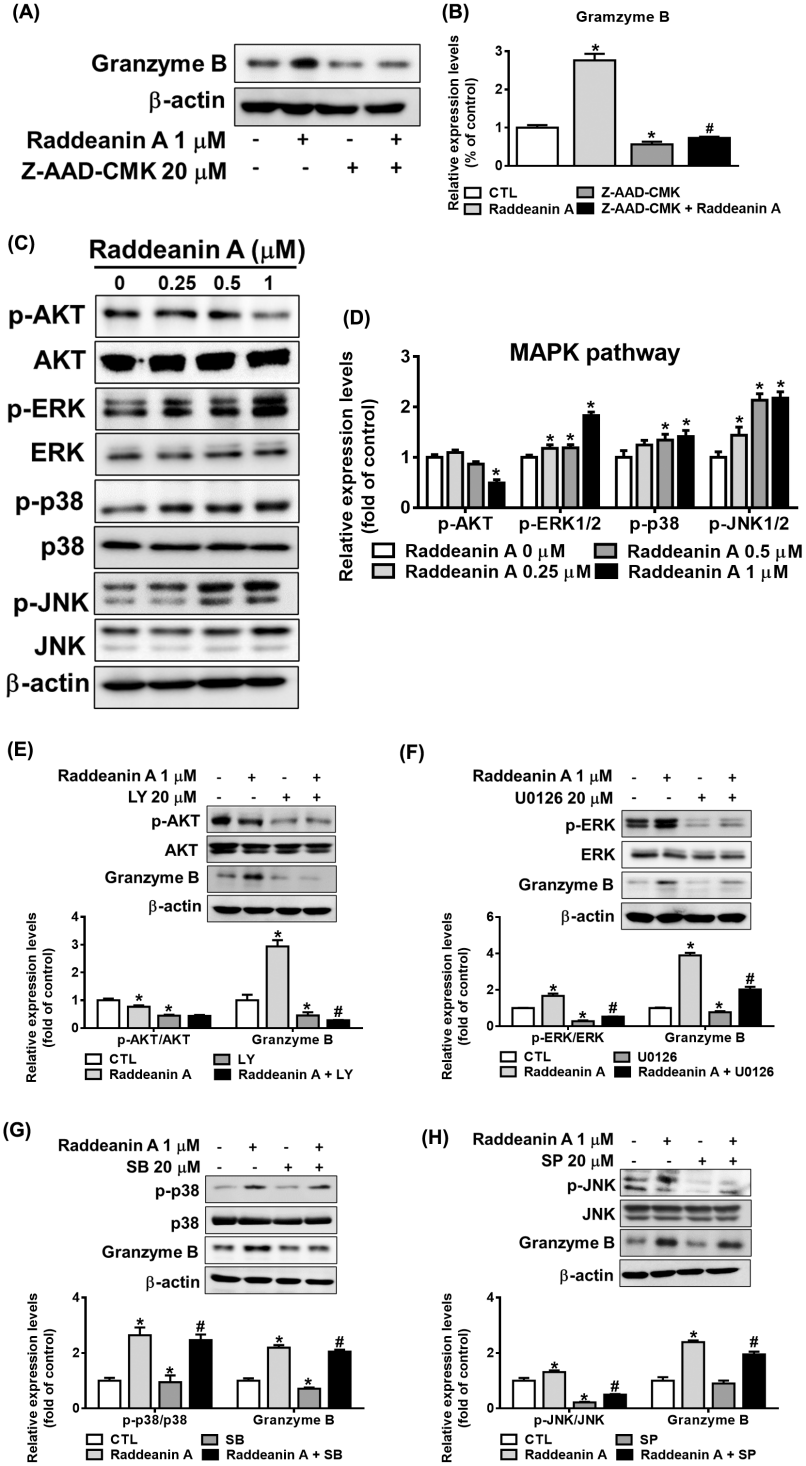
Raddeanin A induces granzyme expression through MAPK pathway in KHYG‐1 cells. (A, B) Cells were pretreated with inhibitor (Z‐AAD‐CMK) for 1 h or left untreated; subsequently, these cells were treated with Raddeanin A for 24 h. Protein levels were quantified through western blotting and were normalized to β‐Actin level using a densitometer. (C) The cells were treated with Raddeanin A for 24 h. (D) Protein levels of AKT phosphorylation/AKT, ERK phosphorylation/ERK, p38 phosphorylation/p38 and JNK phosphorylation/JNK. (E–H) Cells were pretreated with various inhibitors (LY‐294002 (LY)/AKT, U0126/ERK, SB‐203580 (SB)/P38 and SP‐600125 (SP)/JNK) for 1 h or left untreated; subsequently, these cells were treated with Raddeanin A for 24 h. Protein levels were quantified through western blotting and were normalized to β‐Actin level using a densitometer. *p < 0.05 compare with control; #*p* < 0.05 compare with inhibitor‐treated cells.

Raddeanin A treatment increased granzyme B expression in KHYG‐1 cells was inhibited by Z‐AAD‐CMK pretreated cells (Figure 6A,B). We further investigated the effect of Raddeanin A on ERK, p38, JNK and AKT protein expressions level. As shown in Figure 6C,D, Raddeanin A treatment induced ERK, p38 and JNK phosphorylation and decreased the phosphorylation of AKT in KHYG‐1 cells. In order to confirm if Raddeanin A induced the production of granzyme B via the MAPK and AKT signalling pathways, we then pretreated KHYG‐1 cells with various inhibitors, such as LY‐294002, U0126, SP‐600125, or SB‐203580, before treating them with Raddeanin A. Granzyme B expression was decreased in KHYG‐1 cells treated with Raddeanin A and MAPK inhibitors in combination, as seen in Figure 6E–H, in comparison to the Raddeanin A treatment alone. According to our research, Raddeanin A modifies the MAPK signalling pathway to raise granzyme B expression in KHYG‐1 cells.

**FIGURE 7:**
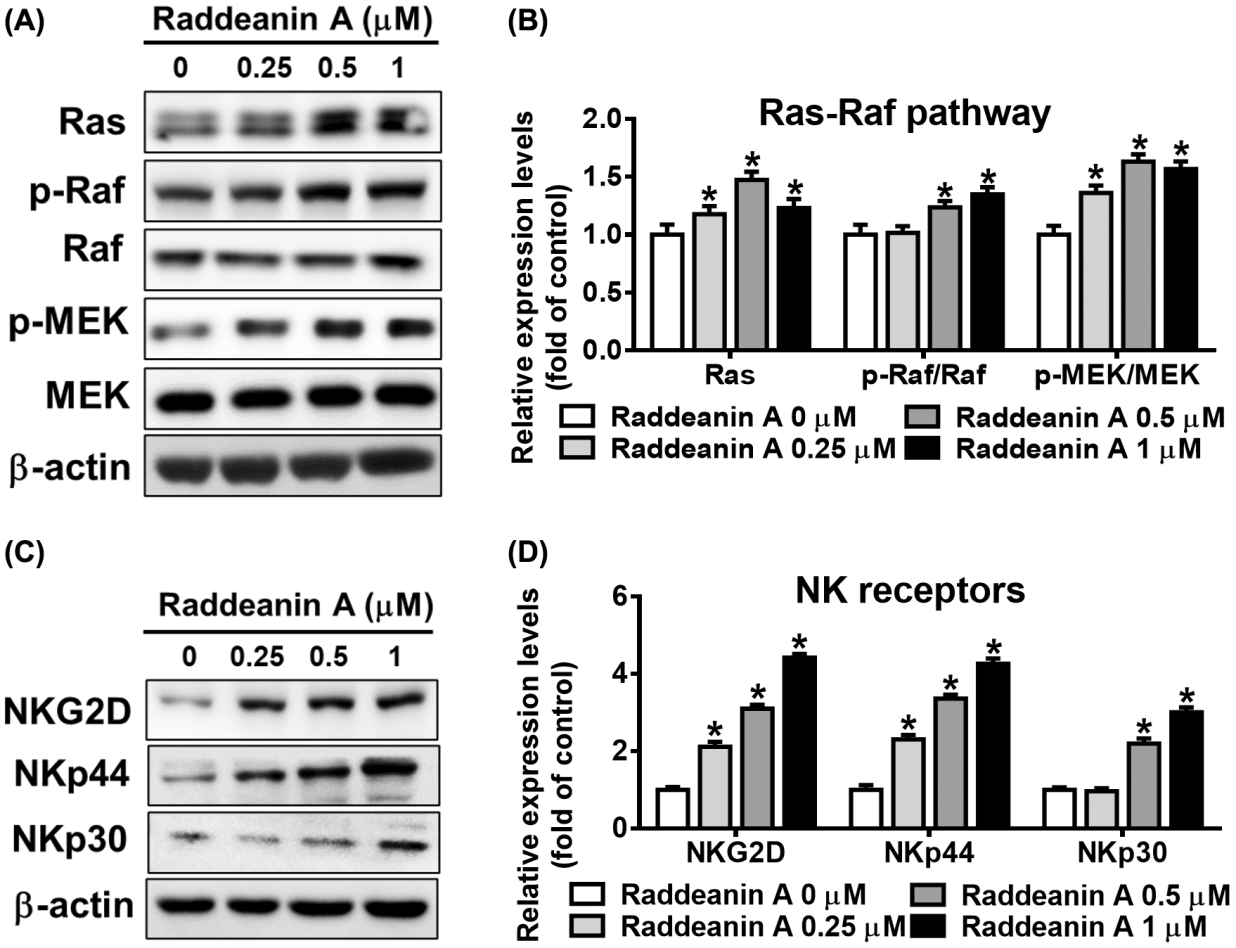
Raddeanin A activates Ras–Raf pathway and receptor expressions in KHYG‐1 cells. (A–D) Effects of Raddeanin A treatment (24 h) on the proteins involved in the Ras, Raf phosphorylation/Raf, MEK phosphorylation/MEK, NKG2D, NKp44 and NKp30 in KHYG‐1 cells. ImageJ was used for protein quantification; the levels of all proteins were normalized to that of β‐Actin. Data are presented as mean ± standard deviation (*n* = 3). *p < 0.05 compare with control.

KHYG‐1 cells were treated with Raddeanin A and then analysed for Ras/Raf/MEK expression. Raddeanin A progressively elevated the Raf and MEK phosphorylation and Ras expression levels in KHYG‐1 cells (Figure 7A,B). We then analysed for activating receptors NKp30, NKp46 and NKG2D in Raddeanin A treated KHYG‐1 cells. Raddeanin A treatment increased NKG2D, NKp44 and NKp30 protein expression compared with the control group (Figure 7C,D).

### 3.5 Raddeanin A triggered Ras/Raf/MEK pathway, leading to increased IFN‐γ expression in NK cells

**FIGURE 8:**
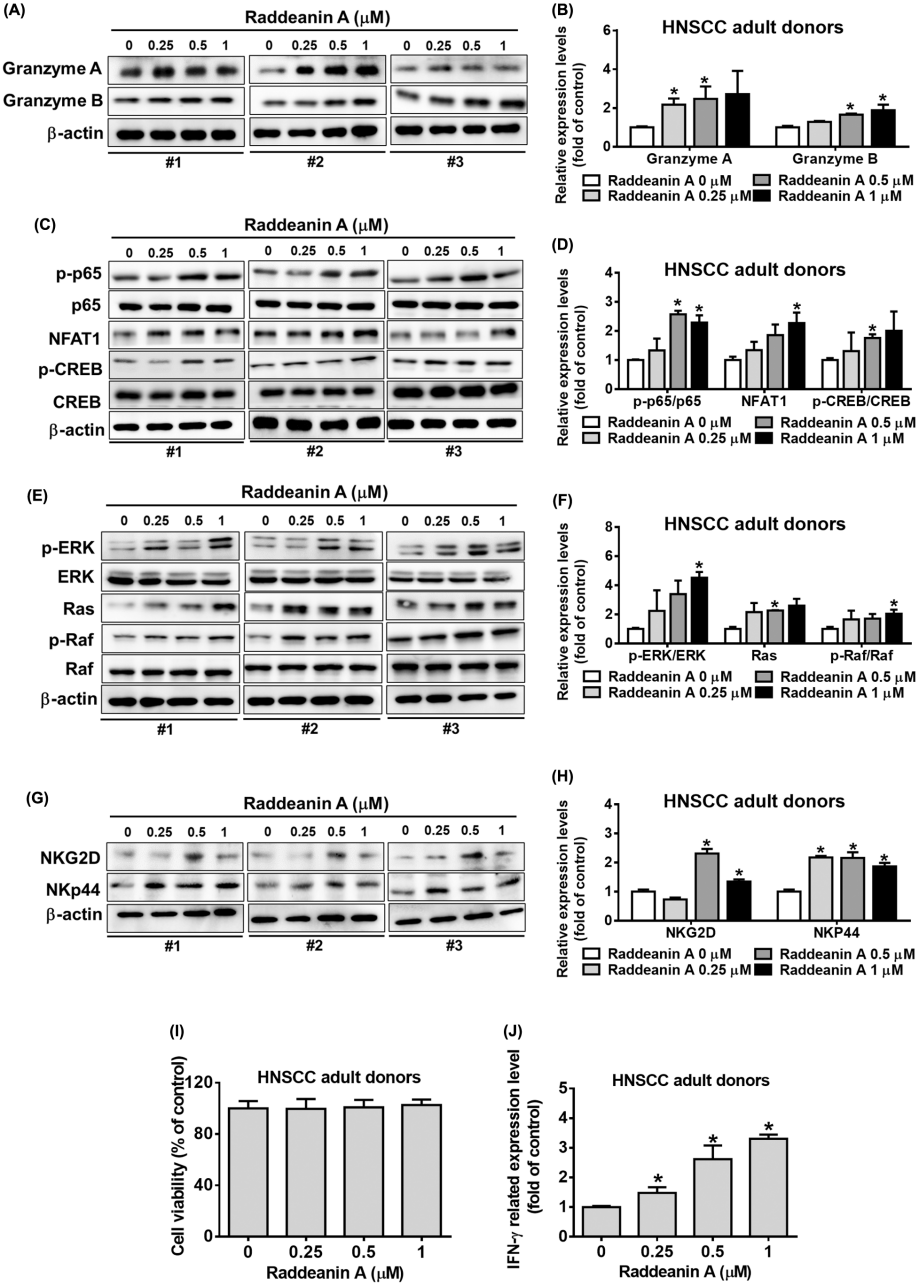
Raddeanin A activates the cytotoxicity effect of NK cells in head and neck cancer patients. (A–H) Effects of Raddeanin A treatment (24 h) on the proteins involved in the granzyme A, granzyme B, p65 phosphorylation/p65, NFAT1, CREB phosphorylation/CREB, ERK phosphorylation/ERK, Ras, Raf phosphorylation/Raf, NKG2D and NKp44 in NK cells obtained from the adult donors with head and neck squamous cell carcinoma (HNSCC). (I) Assessment of cell viability of NK cells treated with Raddeanin A for 24 hours using WST‐8 assay (J) Measurement of IFN‐γ levels in NK cell culture supernatants treated with Raddeanin A for 24 hours using an enzyme‐linked immunosorbent assay (ELISA). ImageJ was used for protein quantification; the levels of all proteins were normalized to that of β‐Actin. Data are presented as mean ± standard deviation (*n* = 3). *p < 0.05 compare with control.

After establishing that Raddeanin A enhanced cytotoxicity in KHYG‐1 cells, we performed the same studies on NK cells derived from adult patients with head and neck squamous cell cancer. As observed in Figure 8A,B, Raddeanin A treatment significantly increased granzymes A and B expressions. Raddeanin A treatment dose‐dependently increased CREB, p65 phosphorylation and NFAT1 protein expression in NK cells (Figure 8C,D). Furthermore, we observed that the Raddeanin A treatment increased ERK, Ras phosphorylation and Raf expression levels in NK cells (Figure 8E,F).

NKG2D and NKp44 protein‐two main activating receptors of NK cells, therefore we verified for this receptor protein expression in NK cells. As shown in Figure 8G,H, Raddeanin A treatment increased NKG2D and NKp44 proteins expression in NK cells. Interactions between these receptors and their ligands are involved in NK‐mediated cytolytic activity and IFN‐γ production.^17^, ^18^ NK cells were treated with Raddeanin A and analysed for cell viability and cytolytic activity. After Raddeanin A treatment did not induce cell viability (Figure 8I), but increased IFN‐γ expression in NK cells (Figure 8J). Our results clearly indicate that Raddeanin A exerts similar effects in both KHYG‐1 cells and NK cells derived from healthy donors.

## 4 DISCUSSION

The present study elucidates the cytotoxic effects of Raddeanin A, a naturally occurring flavonol compound, on KHYG‐1 cells against K562 cells. Raddeanin A enhances the expression of various cytolytic effectors such as perforin, granzymes A and B and FasL through CREB‐mediated pathways. This augmentation enables KHYG‐1 cells to induce mitochondrial membrane depolarization and apoptosis in K562 cells. Furthermore, Raddeanin A induces cell death in K562 cells via both caspase‐dependent (involving t‐Bid, cleaved caspase 3 and cleaved PARP) and caspase‐independent pathways (involving SET, Ape1 and HMGB2). Similar effects have been observed in different cancer cell types. Mechanistically, Raddeanin A enhances ERK, p38 and JNK phosphorylation, leading to increased granzyme B expression in KHYG‐1 cells, consequently facilitating the lysis of K562 cells. Overall, this study underscores the unique role of Raddeanin A in enhancing NK cell cytotoxicity against various cancer cell lines.

To improve NK cell‐based immunotherapy, we investigated how Raddeanin A regulates NK cells. We investigated if Raddeanin A increased IFN‐γ production because it is known that NK cells produce this immune response component. Results showed that Raddeanin A did not affect cell proliferation but increased IFN‐γ production (Figure 1) The extrinsic and intrinsic apoptotic pathways are two in which NK cells take part. By secreting cytotoxic granules containing chemicals such as granzyme, granulysin and perforin into the intercellular space between NK and target cells, they cause the target cells to undergo apoptosis. Granzymes A (gA)^19^ and B (gB),^20^ are the most prevalent among these, prompting apoptosis in target cells via both caspase‐dependent/ independent pathways. Additionally, NK cells generate cytotoxic cytokine‐ IFN‐γ. IFN‐γ modulates members of the tumour necrosis factor‐α cytokine family, including FasL and TRAIL.^21^, ^22^ In this study, Raddeanin A demonstrated a moderate induction of perforin and granzyme A expression. Notably, treatment with Raddeanin A significantly boosted granzyme B and FasL protein expression. These results suggest that Raddeanin A increases the production of cytotoxic effectors, which in turn increases NK cytolytic activity. Particularly, the substantial elevation of granzyme B induced by Raddeanin A appears crucial for augmenting KHYG‐1 cytolytic activity.

Next, we evaluated how Raddeanin A affected the expression of NFAT1, p65 and CREB phosphorylation. Raddeanin A increased CREB and p65 phosphorylation and NFAT1 expression. Furthermore, we investigated whether Raddeanin A influenced chromatin regulation. Our results showed that, Raddeanin A treatment increased histone acetylation; combined treatment with TSA further increased this effect. Our findings are similar to previous findings on nobiletin^23^ and Shuterin^13^‐treated KHYG‐1 cells.

Furthermore, to investigate the impact of Raddeanin A, we employed K562 cells as target cells and KHYG‐1 cells as effector cells. It was observed that KHYG‐1 cells treated with Raddeanin A caused K562 cells mitochondrial membranes to depolarize and increased the number of apoptotic cells. Nevertheless, no change in cell viability was seen when K562 cells were treated with Raddeanin A without also co‐culturing with KHYG‐1 cells (Figure 3F). These findings strongly suggest that K562 cell death is caused by Raddeanin A's cytotoxic effects on KHYG‐1 cells. Raddeanin A induces apoptosis in co‐cultured K562 cells by both caspase‐dependent and caspase‐independent mechanisms, as shown in Figure 3E,G,H. Raddeanin A triggers apoptosis in co‐cultured K562 cells through both caspase‐dependent and independent pathways. We observed comparable mechanisms of action of Raddeanin A against nasopharyngeal carcinoma and squamous cell carcinoma, as depicted in Figures 4 and 5.

In our current investigation, we noted that pretreating with granzyme B inhibitor resulted in the reduction of Raddeanin A‐induced increase in granzyme B expression (Figure 6A,B). Given the substantial impact of Raddeanin A on granzyme B expression, we decided to explore the association of the MAPK pathway in this context. Our results demonstrated that Raddeanin A notably enhanced the phosphorylation of key components of the MAPK pathway, including ERK, p38 and JNK, in KHYG‐1 cells (Figure 6C,D). Furthermore, we observed that co‐administering KHYG‐1 cells with Raddeanin A and MAPK inhibitors significantly attenuated Raddeanin A‐induced induction in granzyme B expression (Figure 6E–H). Raddeanin A notably enhanced the expression levels of Ras, as well as the phosphorylation of Raf and MEK in KHYG‐1 cells. In this study, Raddeanin A elicited Ras expression and the phosphorylation of Raf and MEK, as illustrated in Figure 7A,B. The activating receptors NKp30, NKp46 and NKG2D^24^, ^25^ are present on the surfaces of KHYG‐1 cells. NKp30, NKp44 and NKp46 receptors remain crucial for the strong NK cell activation and NK cell‐mediated destruction of transformed cancer cells.^26^, ^27^ As shown in Figure 7C, NK receptors ‐NKp30, NKp46 and NKG2D were significantly increased after Raddeanin A treatment.

## 5 CONCLUSION

Finally, we noticed that the effects of Raddeanin A were consistent between KHYG‐1 cells and NK cells derived from adult patients with HNSCC. Our findings demonstrated comparable effects of Raddeanin A between KHYG‐1 cells and NK cells from adult patients with HNSCC (refer to Figure 8). In conclusion, Raddeanin A significantly increased IFN‐γ expression, the Ras/Raf signalling pathway and higher NK receptors expression were also observed.

## AUTHOR CONTRIBUTIONS

**Ming‐Ju Hsieh:** Conceptualization (equal); writing – original draft (lead); writing – review and editing (equal). **Jen‐Tsun Lin:** Conceptualization (equal). **Yi‐Ching Chuang:** Methodology (equal); software (equal). **Yu‐Sheng Lo:** Methodology (equal); software (equal). **Chia‐Chieh Lin:** Methodology (equal); software (equal). **Hsin‐Yu Ho:** Methodology (equal); software (equal). **Mu‐Kuan Chen:** Conceptualization (equal); writing – review and editing (equal).

## FUNDING INFORMATION

This research did not receive external funding.

## CONFLICT OF INTEREST STATEMENT

The authors declare no conflicts of interest.

## CONSENT

Informed consent was obtained from all subjects involved in the study.

## INSTITUTIONAL REVIEW BOARD STATEMENT

This study's protocol was approved by the Institutional Review Board (IRB) of the Changhua Christian Hospital (Changhua, Taiwan; IRB no. 230602, date of approval: 11 July, 2023).

## Data Availability

The data used to support the findings of this study are available from the corresponding author upon request.
